# Acetylcytidine modification of DDX41 and ZNF746 by *N*-acetyltransferase 10 contributes to chemoresistance of melanoma

**DOI:** 10.3389/fonc.2024.1448890

**Published:** 2024-08-23

**Authors:** Li Wang, Yuefen Zeng, Ying Zhang, Yun Zhu, Shuangyan Xu, Zuohui Liang

**Affiliations:** ^1^ Tianjin Medical University Cancer Institute and Hospital, National Clinical Research Center for Cancer, Key Laboratory of Cancer Prevention and Therapy, Tianjin’s Clinical Research Center for Cancer, Tianjin, China; ^2^ Department of Dermatology, The People’s Hospital of Yuxi City, Kunming Medical University, Yuxi, Yunan, China; ^3^ Department of Acupuncture and Tuina, The People’s Hospital of Yuxi City, Kunming Medical University, Yuxi, Yunan, China

**Keywords:** melanoma, drug resistance, ac4C-modification, NAT10, C2H2 family

## Abstract

**Background:**

Rapidly developed chemoresistance to dacarbazine (DTIC) is a major obstacle in the clinical management of melanoma; however, the roles and mechanisms of epi-transcriptomic RNA modification in this process have not been investigated.

**Method:**

DTIC-resistant (DR) melanoma cells were established for bulk RNA sequencing. The expressions of mRNAs were detected using qRT-PCR, and protein levels were determined using Western blotting and immunohistochemistry. Acetylated RNAs were detected by dot blotting and immunoprecipitation sequencing (acRIP-seq). A lung metastasis mouse model of melanoma was established to evaluate the anti-melanoma effects *in vivo*.

**Results:**

We identified that the expression of *N*-acetyltransferase 10 (NAT10), a catalytic enzyme for the *N* ^4^-acetylcytidine (ac4C) modification of RNA, was significantly upregulated in the DR cells. Clinically, NAT10 expression was elevated in disease progression samples and predicted a poor outcome. Using ac4C RNA immunoprecipitation (ac4C-RIP), we found that the mRNAs of two C2H2 zinc finger transcriptional factors, *DDX41* and *ZNF746*, were targets of NAT10-mediated ac4C modification. Gain- and loss-of-function experiments in NAT10, or in DDX41 and ZNF746, altered the chemosensitivity of melanoma accordingly, and the two target genes also negatively correlated with clinical outcomes. Finally, pharmacological inhibition of NAT10 with Remodelin sensitized melanoma cells to DTIC treatment *in vitro* and in a mouse xenograft model.

**Conclusion:**

Our study elucidates the previously unrecognized role of NAT10-mediated ac4C modification in the chemoresistance of melanoma and provides a rationale for developing new strategies to overcome chemoresistance in melanoma patients.

## Introduction

Melanoma, a type of skin cancer originating from melanocytes, remains the most lethal among primary cutaneous neoplasms worldwide (1). The incidence of malignant melanoma has steadily increased, yet the median survival of patients has not significantly improved (2). Despite advancements in therapeutic regimens targeting cancer immunology, oncogenic cell signaling pathways, and histone modifications, the prognosis for metastatic melanoma remains poor. Dacarbazine (DTIC) is a commonly used drug for melanoma treatment; however, its long-term survival benefit is extremely limited due to the rapid development of chemoresistance and metastasis, which signify the requirement of new biomarkers for prediction of treatment response, as well as novel medicines that overcome or bypass these resistance mechanisms (3).

Increasing evidence suggests that abnormal regulation of RNA epigenetics plays essential roles in alternative splicing, nuclear export, mRNA stability, and translation efficiency of RNAs, thereby promoting tumor proliferation, metastasis, and maintenance of stemness (4). Moreover, the high frequency and early occurrence of RNA modifications make it an important area of investigation and a potential source of biomarkers and therapeutic targets in cancers (5). For instance, m^6^A is the most abundant mRNA modification, and METTL3, a key component of the m^6^A methyltransferase complex, is considered a potential therapeutic target for several cancers (6). Notably, *N* ^4^-acetylcytidine (ac4C) is the first and sole acetylation modification identified on mRNA, playing a key role in affecting mRNA stability and translation efficiency in tumors (7). *N*-Acetyltransferase 10 (NAT10) is the only human mRNA ac4C modification enzyme with both acetyltransferase and RNA-binding activities and has been proven to be related to the development and prognosis of various cancers (8). For example, NAT10 acetylates *CEP170* mRNA to enhance CEP170 translation efficiency and leads to myeloma growth (9), and NAT10 promotes cisplatin chemoresistance in bladder cancer cells by enhancing DNA damage repair (DDR) (10). Therefore, the therapeutic potential of NAT10 inhibitor Remodelin has been tested in many cancers (11, 12). Nevertheless, whether NAT10 participates in the chemoresistance of melanoma cells remains to be clarified.

In this study, we examined the expression of NAT10 in the DTIC-resistant melanoma cells and screened target genes of NAT10-mediated RNA modification using acetylated RNA immunoprecipitation and sequencing (acRIP-seq). Furthermore, we investigated the mechanism of NAT10 promoting the mRNA ac4C modification of C2H2 zinc finger family members *DDX41* and *ZNF746* in chemoresistance of melanoma and finally evaluated the translational significance of NAT10 inhibitor in overcoming chemoresistance *in vitro* and *in vivo*.

## Materials and methods

### Cell lines

Human melanoma cell lines MeWo and A375 were purchased from ATCC (American Type Culture Collection, Manassas, VA, USA) and were substantiated as mycoplasma-free. MTS assay to detect cell viability was performed as previously described (13, 14). HEK293T cells were a kind gift from Prof. Yupeng Chen at the Department of Biochemistry and Molecular Biology, Tianjin Medical University. Tumor cells were cultured in RPMI-1640 medium supplemented with 10% fetal bovine serum (Gibco, Life Technologies, Carlsbad, CA, USA). The HEK293T cells were cultured in DMEM–high glucose medium with 10% fetal bovine serum (Gibco, Life Technologies, Carlsbad, CA, USA). These cells were all cultured at 37°C in a humidified incubator with 5% CO_2_ (Gibco, Life Technologies, Carlsbad, CA, USA). All cells were authenticated by STR profiling (Biowing Biotech, Shanghai, China) and confirmed to be mycoplasma-free using the Universal Mycoplasma Detection Kit (ATCC, Manassas, VA, USA).

### Transfection, virus package, infection, and luciferase assay

HEK293T cells were transfected using polyethyleneimine (PEI) (Polysciences, Warrington, PA, USA) in OPTI-MEM medium (Life Technologies, Carlsbad, CA, USA) with a DNA : PEI ratio of 1:4 to 1:6. Viral particles were produced by HEK293T cells in a 10-cm dish transfected with 4 μg PMD2G and 6 μg PSPAX2 packaging plasmids, along with 8 μg lentiviral expressing vectors. The supernatant carrying the viral particles was harvested after transfection and concentrated to 1/100th of the original volume using poly(ethylene glycol) 8000 (Sigma-Aldrich, St. Louis, MS, USA). For viral infection, melanoma cells were seeded in a 6-well-plate, and an increasing dosage of viral concentration and 8 μg/mL polybrene was added. Twelve hours after infection, the medium was changed, and cells were cultured for another 48 hours for further experiment.

### Real-time PCR

Total RNA was isolated from cells using TRIzol (Life Technologies, South San Francisco, CA, USA) according to the manufacturer’s instructions. Total RNA was reverse transcribed using the 5× All-In-One Reverse Transcription MasterMix (abm, Vancouver, Canada). Quantitative real-time PCR was performed by mixing cDNA, gene-specific primers, and EvaGreen 2× qPCR MasterMix (abm, Vancouver, Canada) in the QuantStudio 3 Real-Time PCR System (Applied Biosystems). The primers used in qPCR are listed in the **Supplementary Material**.

### Cell viability assays

For Cell Counting Kit-8 (CCK-8, APExBIO Technology) assays, cells were seeded at 1 × 10^5^ cells/well in 96-well plates; then treated with DTIC, Remodelin, or dimethyl sulfoxide (DMSO); and incubated at 37°C in 5% CO_2_ for 48 hours. CCK-8 reagent was added to each well according to instructions and incubated for 1 hour before reading absorbance at 450 nm. Percentage = OD value of the treatment group/OD value of the control group × 100.

### DTIC-resistant cell induction

A375 and MeWo melanoma cells were a kind gift from Prof. Tao Li at Hunan Normal University, School of Life Sciences. The DTIC resistance phenotype was achieved after exposure of A375 and MeWo cells to increasing concentrations of DTIC (Libbs, Sao Paulo, SP) for 3 months using a modified method according to Marinello et al. (15). Viable cells after the last passage were considered dacarbazine-resistant (DR).

### Xenografts and lung metastasis model

A total of 1 × 10^6^ A375 cells were re-suspended in Matrigel (Becton Dickinson) and subcutaneously injected into lateral flanks of adult female NOD/SCID mice. Mice were randomly chosen for the two experimental groups and housed in specific pathogen-free conditions. Subcutaneous tumor size was blindly measured twice a week using a caliper. Tumor volumes were calculated using the formula V = W^2^ × L × 0.5, where W is tumor width and L is tumor length. For the lung metastasis model, 1 × 10^6^ A375 cells were intravenously inoculated to the tail vein of SCID mice. After 3 weeks, mice were sacrificed, and lungs were removed for lung metastasis analysis.

### TUNEL assay

TUNEL assay was performed using the Deadened™ Fluorometric TUNEL System (Promega, Tokyo, Japan). For paraffin-embedded sections, slides were washed three times with 100% ethanol for 15 minutes at room temperature and then rehydrated by sequentially immersing the slides through graded ethanol washes (95%, 85%, 70%, and 50%) for 3 minutes each at room temperature. The slides were further washed in phosphate-buffered saline (PBS) three times at room temperature and incubated with 100 μL of 20 μg/mL Proteinase K for 30 minutes at room temperature. They were then washed and incubated with rTdT incubation buffer at 37°C for 60 minutes in the dark. The samples were washed and stained with DAPI for 5 minutes at room temperature in the dark, then washed three times, and analyzed using the Olympus FV1000 IX81-SIM Confocal Microscope (Olympus, Tokyo, Japan).

### Western blotting

Cells were placed in a culture dish on ice and washed with ice-cold PBS, after which ice-cold RIPA buffers with protease inhibitors (Roche, Indianapolis, IN, USA) were added. Ice-cold temperatures were maintained at 4°C for 30 minutes, after which the cells were centrifuged in a microcentrifuge for 20 minutes at 12,000 rpm at 4°C. A small volume of lysate was removed to perform a protein quantification assay and adjust the sample concentration. Equal amounts of protein were loaded into the wells of the sodium dodecyl sulfate–polyacrylamide gel electrophoresis (SDS-PAGE) gel, along with the molecular weight marker. The gel was run for 1–2 hours at 100 V, and the protein was transferred from the gel to the nitrocellulose membranes (Pall Corporation, Washington, NY, USA). The membrane was blocked for 1 hour at room temperature and then incubated with the indicated antibody in a blocking buffer at 4°C overnight. The membrane was washed in PBST three times, incubated with horseradish peroxidase-conjugated secondary antibodies at room temperature for 1 hour, and finally visualized using an enhanced chemiluminescence system (Millipore, Los Angeles, CA, USA). The representative Western blotting images from at least three independent experiments shown in the figures were cropped and auto-contrasted. Quantifications of Western blotting were analyzed using ImageJ Version 1.53c (National Institutes of Health).

### Immunohistochemistry

To block deparaffinized tissue slides, a 3% H_2_O_2_ solution was used, and citrate buffer (pH 6.0) was used to retrieve the antigen. After successful blocking of the deparaffinized tissue, appropriately diluted primary antibodies were added onto the slides and incubated in a humidified chamber at 4°C overnight, after which diluted biotinylated secondary antibodies were incubated for 1 hour at room temperature. DAB substrate solution (Dako, K5361), which was newly made just before use, was utilized to reveal the color of antibody staining. Hematoxylin staining was used to localize Nuclei for 1 to 2 minutes before mounting and capture.

### Flow cytometry analysis and cell viability assays

Melanoma cells were incubated in 6-well plates and administered with DTIC with or without Remodelin; then, cells were collected and washed twice with PBS; apoptosis assay was then carried out using the Annexin V-FITC Apoptosis Detection Kit (Sigma-Aldrich, St. Louis, MO, USA) according to the manufacturer’s instructions. A total of 1 × 10^6^ cells were stained with 10 μL Annexin V-FITC and 2 μL of propidium iodide (PI) in the dark to ensure the population abundance. Analysis was performed on the CellQuest 3.0 software (BD Biosciences, New Jersey, USA) and interpreted using the FlowJo software (Treestar).

### Ac4C dot blot

Total RNA was heated to 65° for 5 minutes, immediately placed on ice for 1 minute, and loaded onto Hybond-N+ membranes. Membranes were crosslinked with 150 mJ/cm^2^ in a UV254 nm Stratalinker 2400 (Stratagene). Then, membranes were blocked with 5% non-fat milk for 1 hour at room temperature and incubated with an anti-ac4C antibody (ab252215; Abcam) in PBST (1:1000) at 4° overnight. The membranes were then washed three times with 0.1% PBST and incubated with 0.02% methylene blue solution for 10 minutes, and the background was rinsed with diethylpyrocarbonate (DEPC)-treated water and scanned as an internal reference.

### Acetylated RNA immunoprecipitation and sequencing

NAT10-OE and Vec A375 cells were used for acRIP-seq analysis. The procedure of acRIP-seq was performed as previously described (7). For each IP, 2.5 mg of anti-NAT10 antibody or 2.5 mg of rabbit IgG control was pre-coupled to 300 μg Protein G Dynabeads (Thermo Fisher Scientific) in PBS for 1 hour at room temperature. DNase-treated total RNA was immunoprecipitated for 4 hours at 4°C in 100 μL of acRIP buffer and anti-ac4C or IgG pre-coupled Protein G Dynabeads. After immunoprecipitations, beads were washed five times in acRIP buffer, and elution of RNA was carried out by digestion. The library was constructed using an EpiTM Mini LongRNA-seq Kit (E1802; Epibiotek) according to the manufacturer’s protocol. Quality control of the library was conducted using a BiOptic Qsep100 Analyzer (BiOptic). NovaSeq, a high-throughput sequencing platform, was used for sequencing by Epibiotek (Guangzhou, China).

### Statistical analysis

Data are shown as mean ± SD for at least three independent experiments. Differences between groups were determined using paired two-tailed Student’s *t*-test or two-way ANOVA. Pearson’s correlation test was used to determine the correlations between gene expressions, and survival analysis was conducted using GraphPad Prism 5.0. A *p*-value of less than 0.05 was considered statistically significant (*p ≤ 0.05 and **p ≤ 0.01, compared with the controls).

## Results

### High NAT10 expression is correlated with DTIC resistance and poor clinical outcome of melanoma

**Figure 1:**
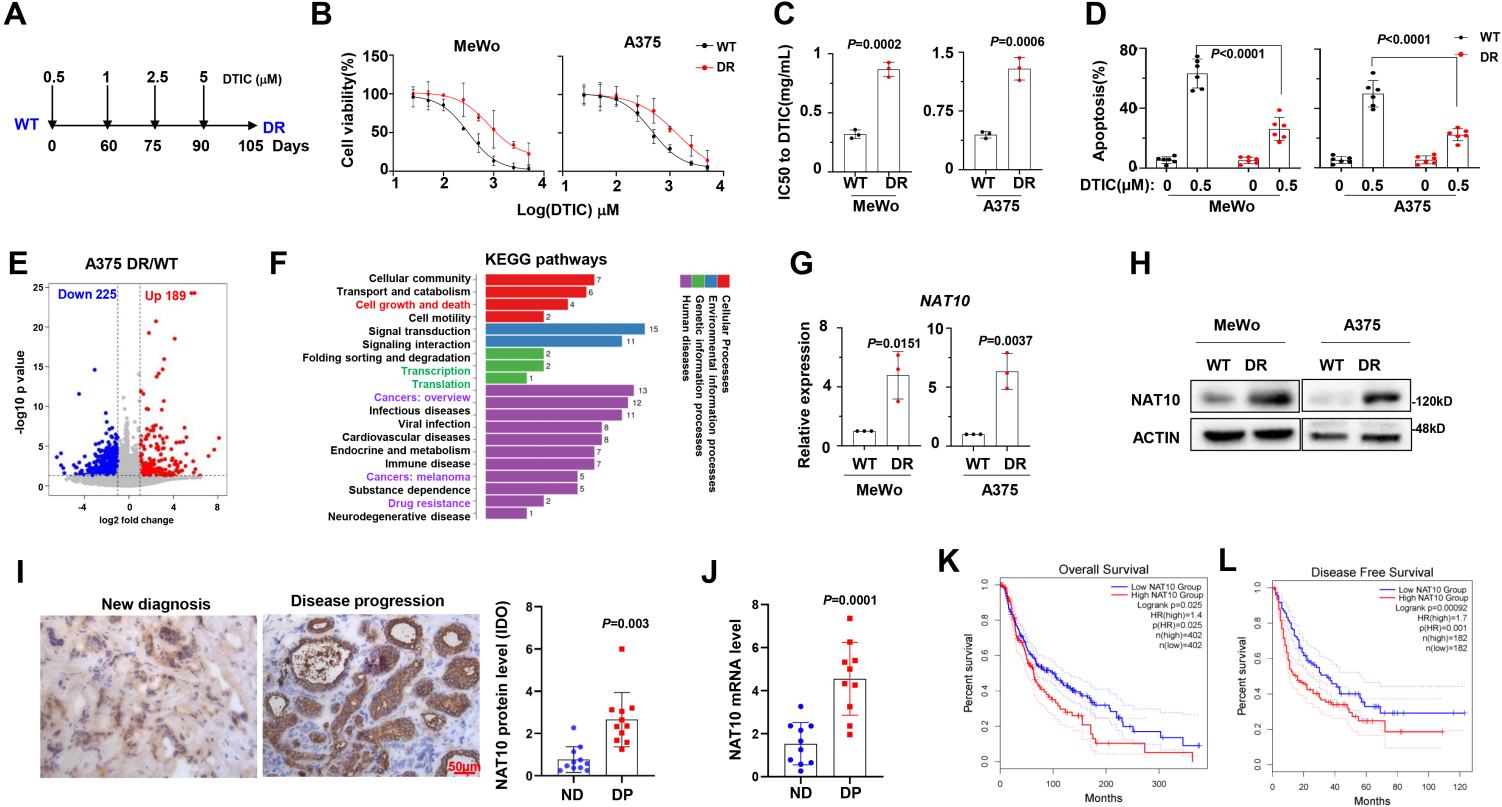
*N*-Acetyltransferase 10 (NAT10) expression is correlated with dacarbazine resistance in melanoma cells. **(A)** Diagram of induction of dacarbazine (DTIC) resistance in human melanoma cell lines. Cells were exposed to increasing concentrations of DTIC 0.5 µg to 5 µg/mL DTIC for 3 months. **(B)** Alteration of IC_50_ to DTIC treatment in the wild-type (WT) and DTIC-resistant (DR) MeWo and A375 cells. **(C)** Comparison of the IC_50_ values of WT and DR cells. **(D)** Flow cytometry analysis of apoptosis of WT and DR cells after DTIC treatment. **(E)** Volcano plot describing genes with upregulated (red) and downregulated (blue) mRNA acetylation levels upon NAT10 overexpression. **(F)** Kyoto Encyclopedia of Genes and Genomes (KEGG) analysis highlighted the Cell growth and death, Transcription, Translation, and Drug resistance pathways in the DR melanoma cells. NAT10 mRNA **(G)** and protein **(H)** expression in WT and DR MeWo and A375 cells. **(I)** Representative immunohistochemical staining and quantification for NAT10 protein in the melanoma tissue slides from newly diagnosed patients and patients with disease progression. Scale bar, 50 µm. **(J)** mRNA levels in newly diagnosed patients and patients with disease progression. **(K, L)** Correlation of *NAT10* mRNA expression with overall survival and disease-free survival in melanoma patients after receiving DTIC-based treatment regimens. Two-sided *p*-values were determined by two-way ANOVA test; mean ± SD. Data representative of n = 3 biologically independent experiments unless stated otherwise.

To investigate the features of DTIC-resistant melanoma cells, we established two DR melanoma cell lines, MeWo and A375, using a method described in a previous report (**Figure 1A**) (15). After 3 months of induction, we examined the sensitivity to DTIC and found that the IC_50_ of DR melanoma cells was significantly increased compared to the wild-type controls (WT) (**Figures 1B, C**). Additionally, DTIC-induced apoptosis, as shown by the flow cytometry assays, was also reduced in the DR cells (**Figure 1D**). To identify the mechanisms underlying DTIC resistance in melanoma cells, we performed bulk cell RNA sequencing in the WT and DR A375 cells, identifying 189 upregulated and 225 downregulated genes in the DR cells (**Figure 1E**). Kyoto Encyclopedia of Genes and Genomes (KEGG) analysis indicated that pathways related to Cell growth and death, Transcription, Translation, and Drug resistance were enriched in the DR cells (**Figure 1F**). Notably, we found that NAT10 was one of the most highly expressed genes in the DR cells, and the expression pattern of NAT10 was also confirmed at the protein and mRNA levels (**Figures 1G, H**). Clinically, we measured NAT10 protein and mRNA levels in patient samples and found that NAT10 expression was significantly elevated in patients with disease progression (**Figures 1I, J**). Remarkably, higher expression of *NAT10* predicted worse overall survival (OS) (**Figure 1K**) and disease-free survival (**Figure 1L**). These results suggest that aberrant expression of NAT10 is closely correlated with melanoma progression.

### NAT10 associated with DTIC sensitivity in melanoma cells

**Figure 2:**
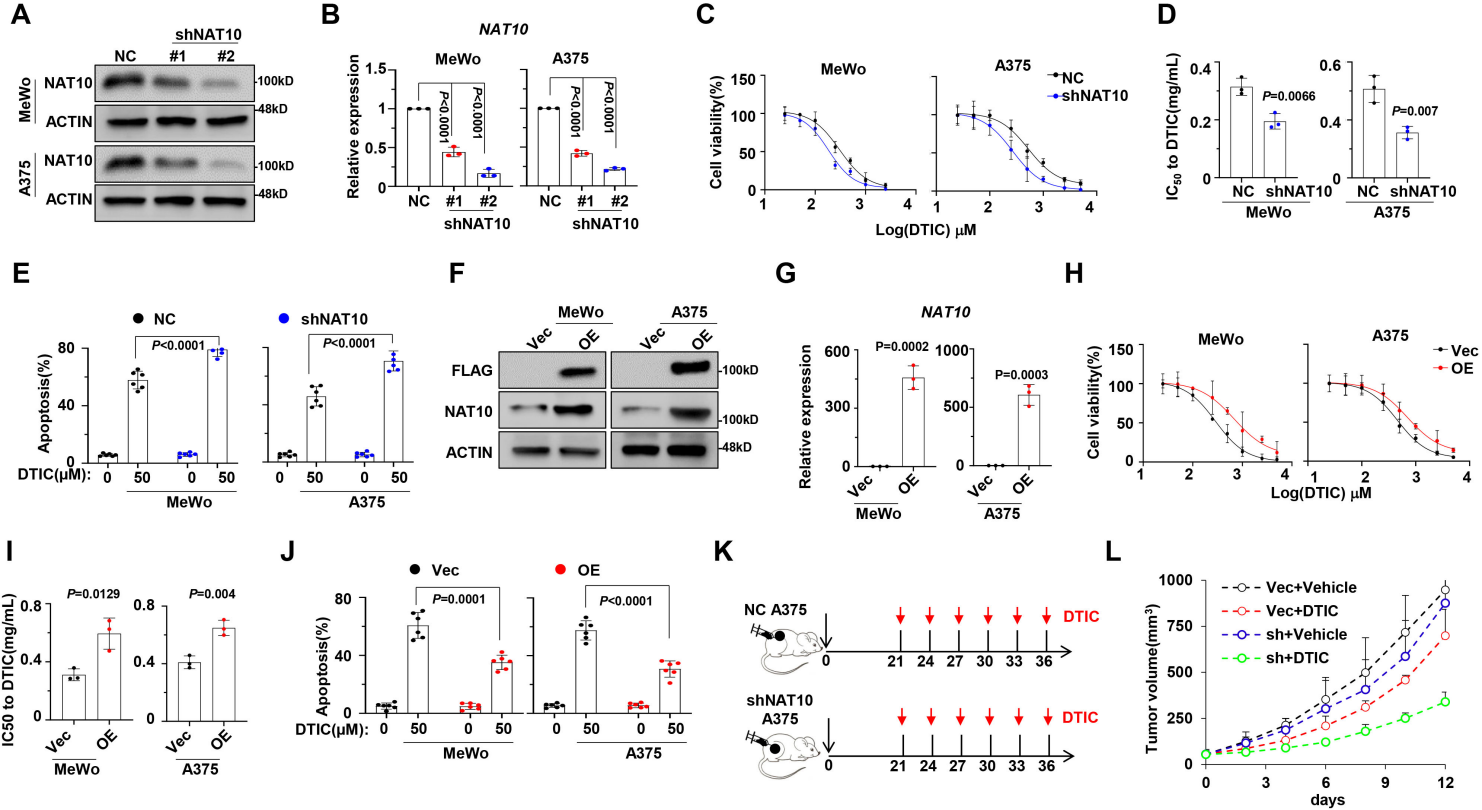
Manipulation of *N*-acetyltransferase 10 (NAT10) expression alters sensitivity to dacarbazine (DTIC) treatment *in vitro* and *in vivo*. **(A)** Representative Western blotting shows the knockdown effects in MeWo and A375 cells infected with lentivirus carrying three shRNAs targeting two different coding sequencing of NAT10 gene (shRNA1 and shRNA2) compared to the non-target control (NT Ctrl). **(B)** Histogram showing *NAT10* relative expression of shNAT10 and NC MeWo and A375 cells. **(C)** Alteration of IC_50_ to DTIC treatment in the NT Ctrl (NC) and NAT10 knockdown (shNAT10) cells; **(D)** comparison of the IC_50_ values of NC and shNAT10 cells. **(E)** Frequency of apoptosis cells after DTIC treatment in WT cell. **(F)** Representative Western blotting shows the ectopic expression of NAT10 in MeWo and A375 cells infected with lentivirus carrying the NAT10-flag (NAT10-OE) compared to the vector control. **(G)** Histogram showing *NAT10* relative expression of shNAT10 and NC MeWo and A375 cells. **(H)** Alteration of IC_50_ to DTIC treatment in the Vec and NAT10-OE cells and **(I)** comparison of the IC_50_ values of Vec and NAT10-OE cells. **(J)** Frequency of apoptosis cells after DTIC treatment. **(K)** Experimental setup used to assess the effects of NAT10 on melanoma tumor growth and DTIC sensitivity *in vivo* in NOD/SCID mice. Mice were s.c. injected with A375 NC or shNAT10 cells followed by i.v. injections of DTIC or phosphate-buffered saline (PBS) every 3 days. **(L)** Tumor growth was measured and calculated as 1/2(L × W^2^) mm, where L presents the length and W represents width of tumor. Relative tumor growth curves of A549 tumors. Two-sided *p*-values were determined by two-way ANOVA test; mean ± SD. Data representative of n = 3 biologically independent experiments unless stated otherwise.

To further determine the effects of NAT10 on drug resistance of melanoma cells, we inhibited the expression of NAT10 using short-hairpin RNA (shRNA) and selected shRNA #2 for subsequent experiments due to its highest knockdown efficacy. As expected, the expression levels of NAT10 in MeWo and A375 cells were significantly suppressed via shRNA transfection (NAT10-KD) (**Figures 2A, B**). Consequently, the IC_50_ values for DTIC were significantly reduced in the NAT10-KD cells (**Figures 2C, D**), accompanied by a marked increase in apoptotic cells (**Figure 2E**). However, ectopic expression of NAT10 in MeWo and A375 cells (**Figures 2F, G**) remarkably augmented the resistance to DTIC treatment (**Figure 2H**) and abrogated DTIC-induced melanoma apoptosis, as evidenced by the increased IC_50_ value and decreased apoptosis rate (**Figures 2I, J**). To further evaluate the effects of NAT10 on melanoma tumor growth and DTIC sensitivity *in vivo*, we established a NAT10-KD or WT A375 cell-derived xenograft mouse model of melanoma in NOD/SCID mice and then treated it with DTIC or vehicle control every 3 days (**Figure 2K**). Analyzing tumor sizes after treatment, we observed that suppression of NAT10 in melanoma cells inhibited tumor growth and sensitized A375 cells to DTIC treatment (**Figure 2L**). Overall, these data coherently suggest that targeting NAT10 contributed to overcoming DTIC resistance of melanoma cells *in vitro* and *in vivo*.

### NAT10-mediated *N* ^4^-acetylcytidine of *DDX41* and *ZNF746* mRNA

**Figure 3:**
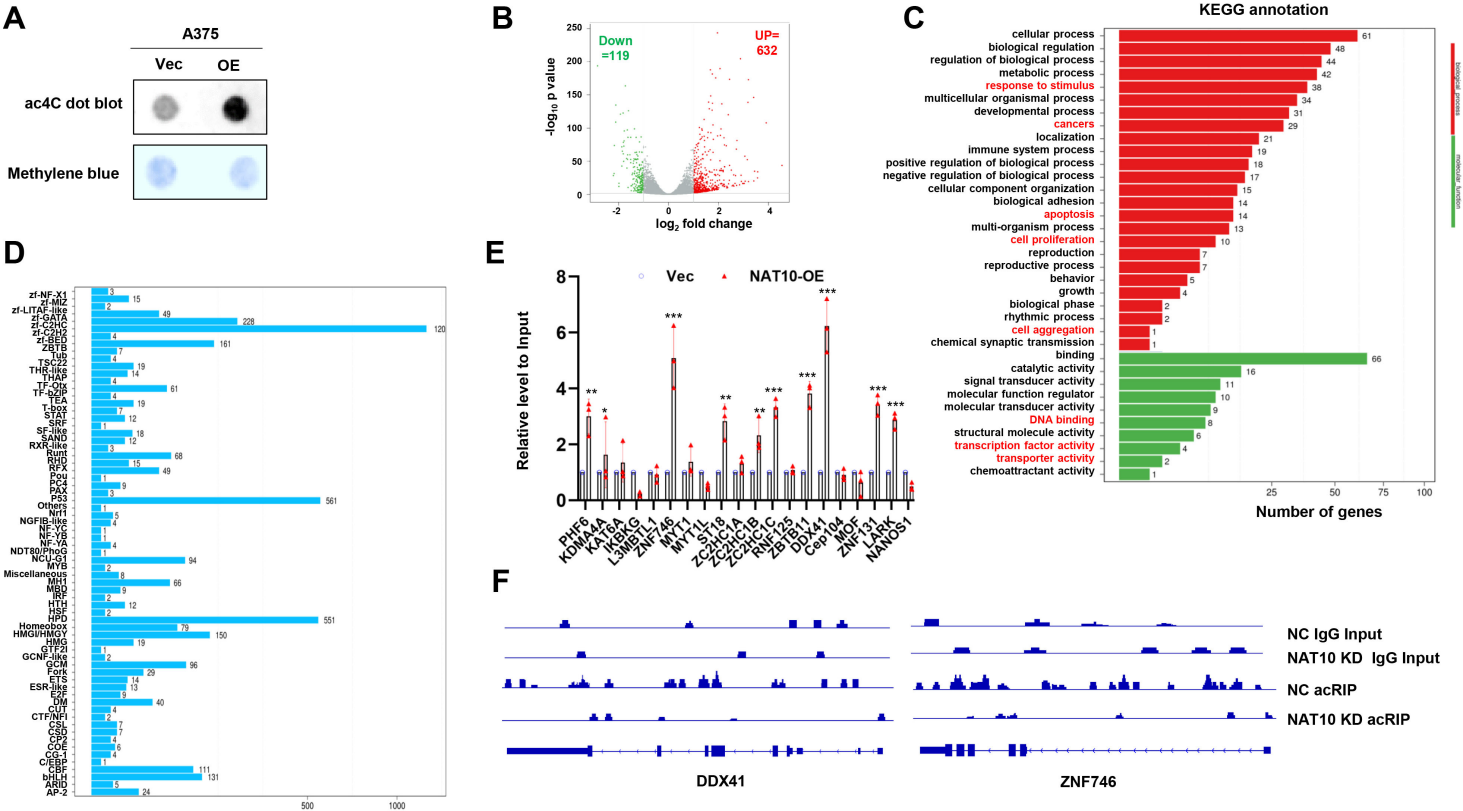
*N*-Acetyltransferase 10 (NAT10) acetylates mRNA to regulate gene expression of melanoma cells. **(A)** Dot blot assay showing that increased NAT10 led to higher mRNA acetylation levels. **(B)** Volcano map of differentially expressed ac4C gene peaks upon NAT10 overexpression. **(C)** Kyoto Encyclopedia of Genes and Genomes (KEGG) analysis of acRIP-seq. **(D)** Genes with *N* ^4^-acetylcytidine (ac4C) modification were enriched in C2H2 zinc finger genes (ZF-C2H2) family. **(E)** Detection of ZF-C2H2 family gene relative expression in NAT10-OE and Vec melanoma cells. **(F)** Gene tracks showing representative acRIP-Seq profiles at *DDX41* and *ZNF746* gene loci in NAT10-KD and NC A375 cells. Two-sided *p*-values were determined by two-way ANOVA test; mean ± SD. Data representative of n = 3 biologically independent experiments unless stated otherwise. *p < 0.05, **p < 0.01, and ***p < 0.001.

Since NAT10 is an acetyltransferase, we investigated whether its promotion of melanoma cell chemoresistance is dependent on its acetyltransferase activity. We measured mRNA acetylation levels in melanoma cells with either forcibly expressed NAT10 or vector control, and the ac4C level of mRNAs in the NAT10-OE cells was obviously increased (**Figure 3A**). To define the targets of NAT10, we performed the acRIP-seq assay in NAT10-OE and Vec-A375 cells to assess the ac4C distribution. AcRIP-seq identified 632 genes with increased ac4C modification and 119 genes with decreased ac4C modification in NAT10-OE A375 cells compared to Vec cells (**Figure 3B**). KEGG analysis revealed that mRNA acetylated genes were correlated with cell proliferation, DNA binding, transcription activity, and so on (**Figure 3C**), and 120 genes belonging to the Cys2His2 zinc finger (ZF-C2H2) family (**Figure 3D**). ZF-C2H2 genes constitute the largest class of transcription factors in humans, and many ZF-C2H2 proteins can promote cancer progression (16). This led us to hypothesize that NAT10 promotes melanoma malignancy by regulating ZF-C2H2 gene transcription. Therefore, we further screened candidate ZF-C2H2 genes and found that *DDX41* and *ZNF746* were the most significantly upregulated genes in NAT10-OE cells (**Figure 3E**). In addition, ac4C peaks of *DDX41* and *ZNF746* demonstrated significantly decreased enrichment in NAT10-KD cells (**Figure 3F**). These data implicate that *DDX41* and *ZNF746* are downstream targets of NAT10 in melanoma.

### DDX41 and ZNF746 correlate with chemosensitivity of melanoma

**Figure 4:**
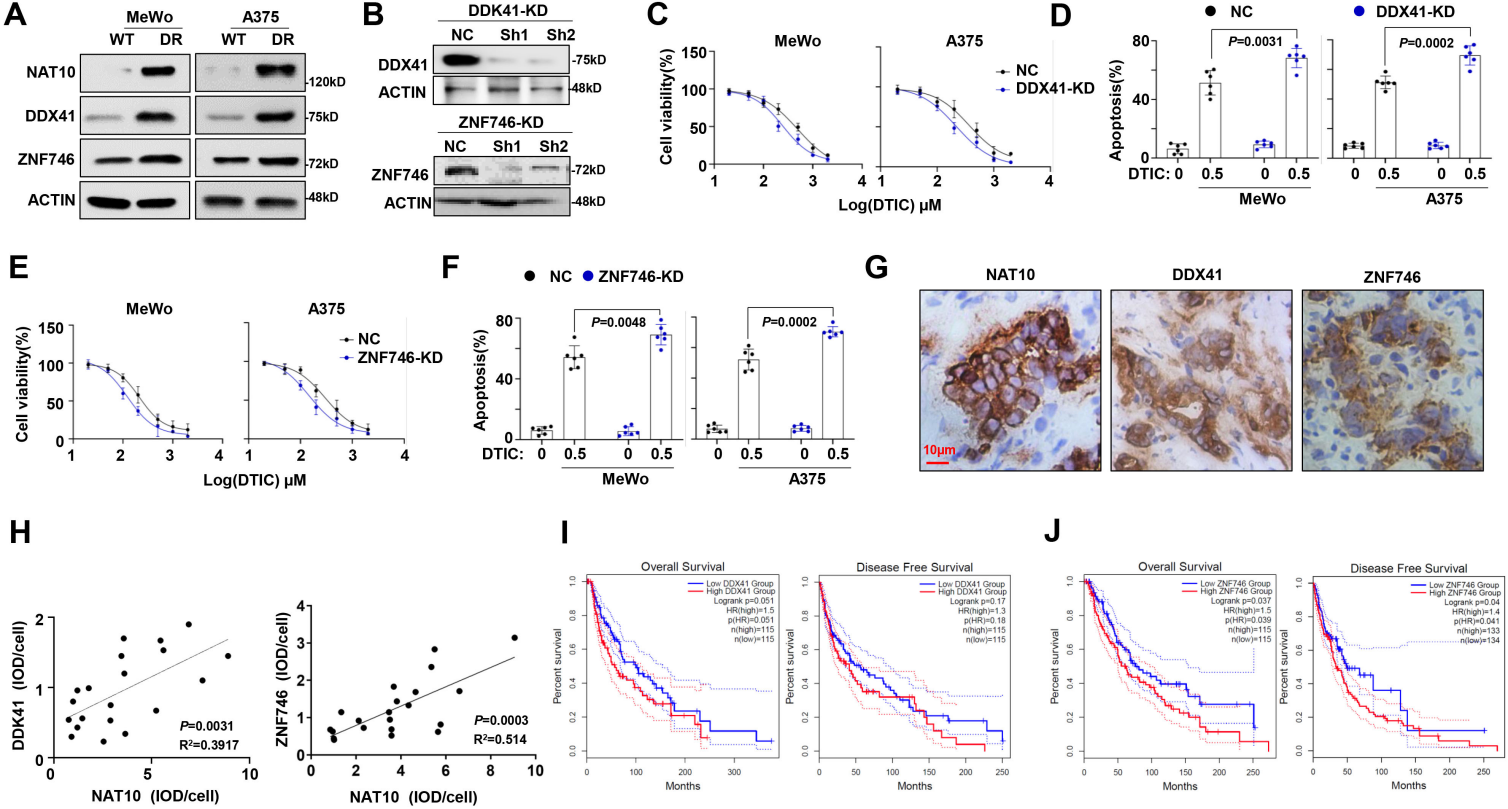
DDX41 and ZNF746 expression correlated with dacarbazine (DTIC) resistance in melanoma cells. **(A)** Protein level of DDX41 and ZNF746 after induction of DTIC resistance in MeWo and A375 cells. **(B)** Representative Western blotting shows the knockdown effects in A375 cells infected with lentivirus carrying three shRNAs targeting two different coding sequencing of DDX41 and ZNF746 gene (shRNA1 and shRNA2) compared to the non-target control (NT Ctrl). **(C)** Alteration of IC_50_ to DTIC treatment in the NT Ctrl (NC) and DDX41 knockdown (KD) cells. **(D)** Flow cytometry analysis of cell apoptosis of DDX41-KD and NC melanoma cells induced by 0.5 μg/mL DTIC for 48 hours. **(E)** Alteration of IC_50_ to DTIC treatment in the NT Ctrl (NC) and ZNF746-KD cells. **(F)** Flow cytometry analysis of cell apoptosis assay of ZNF746-KD and NC melanoma cells induced by 0.5 µg/mL DTIC for 48 hours. **(G)** Representative immunohistochemical staining for *N*-acetyltransferase 10 (NAT10), DDX41, and ZNF746 protein in the melanoma tissue slides from same melanoma patient show the correlation of expression. Scale bar, 10 µm. **(H)** Correlation of NAT10 with DDX41 expression; NAT10 and ZNF746 expression in clinical samples of melanoma patients (n = 20). **(I, J)** Correlation of *DDX41* and *ZNF746* expression with progression-free survival (PFS) and overall survival (OS) in melanoma patients after receiving DTIC-based treatment regimens. *p*-Values were determined by two-way ANOVA test, Pearson’s coefficient, and log-rank test. Data representative of n = 3 biologically independent experiments unless stated otherwise.

Given that DDX41 and ZNF746 are downstream targets of NAT10, we aimed to detect their role in DTIC sensitivity. We measured the protein levels of DDX41 and ZNF746 in our established DR melanoma cells, revealing that increased NAT10 was correlated with elevated DDX41 and ZNF746 proteins (**Figure 4A**). We further suppressed the expression of DDX41 and ZNF746 using shRNA, used DDX41 #Sh1 and ZNF746 #Sh1 for subsequent experiments (**Figure 4B**), and observed that the IC_50_ values of DTIC were meaningfully suppressed (**Figures 4C, E**). Furthermore, knockdown of DDX41 and ZNF746 sensitized melanoma cells to DTIC treatment (**Figures 4D, F**). Remarkably, immunohistochemical staining for NAT10, DDX41, and ZNF746 protein on tumor tissues from melanoma patients showed that NAT10 had a positive correlation with these two proteins (**Figures 4G, H**). Follow-up analysis also indicated that DDX41 and ZNF746 expressions were negatively correlated with OS and progression-free survival (PFS) of melanoma patients (**Figures 4I, J**). Collectively, these data suggest that DDX41 and ZNF746 are correlated with NAT10 expression and play a pivotal role in the treatment response and clinical outcomes of melanoma patients.

### Targeting NAT10 enhanced chemosensitivity of melanoma *in vitro* and *in vivo*

**Figure 5:**
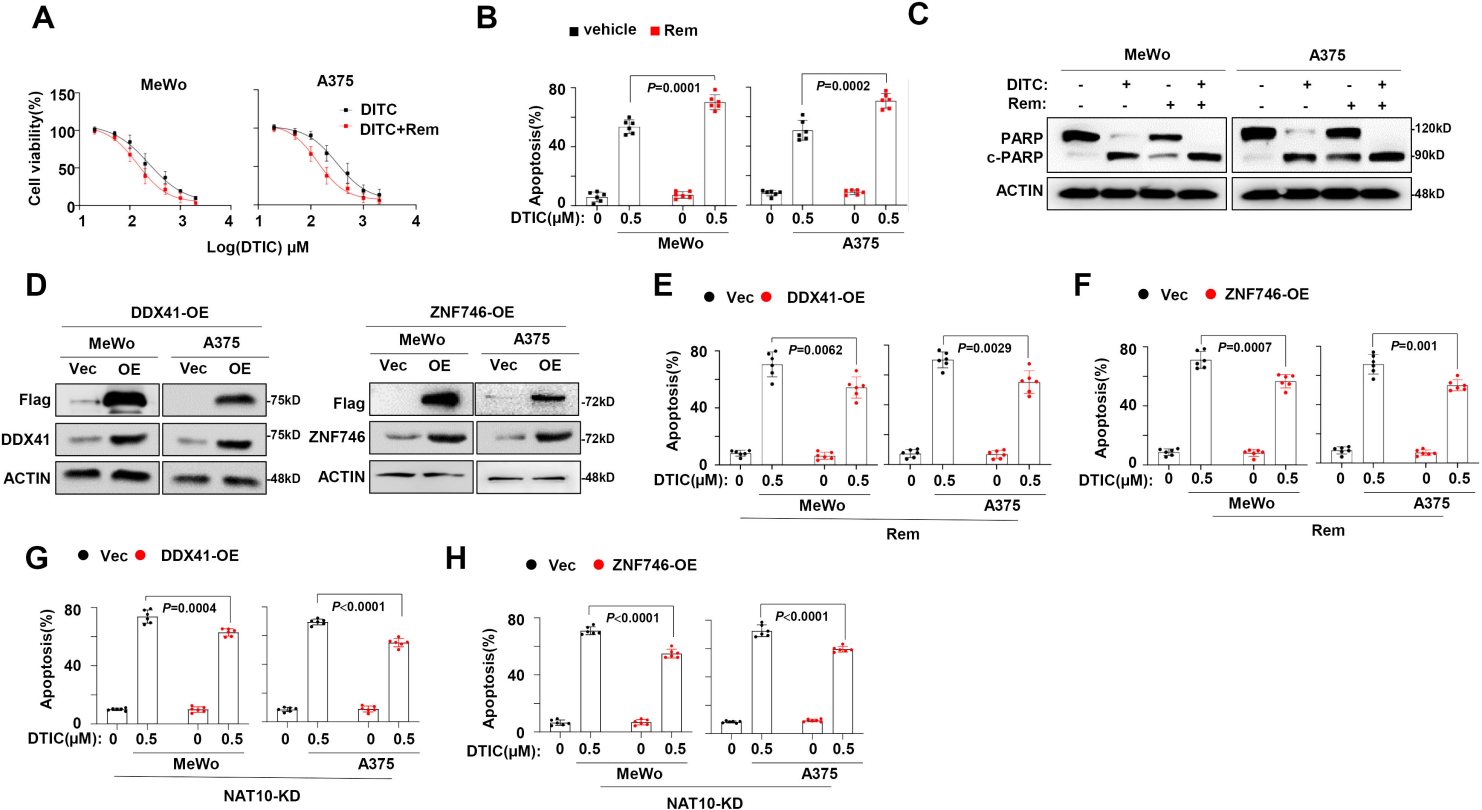
*N*-Acetyltransferase 10 (NAT10) promotes melanoma cells’ dacarbazine (DTIC) resistance through DDX41 and ZNF746. **(A)** NAT10 inhibitor Remodelin alters the IC_50_ to DTIC treatment in DTIC-resistant (DR) MeWo and A375 cells. **(B)** Flow cytometry analysis of cell apoptosis of DR MeWo and A375 cells induced by 0.5 µg/mL DTIC for 48 hours. **(C)** Western blotting analysis of the cleavage of PARP showed that DTIC and Remodelin synergetically promote melanoma cell apoptosis.**(D)** DDX41 and ZNF746 protein expression of MeWo and A375 cells infected with lentivirus carrying DDX41-Flag or ZNF746-Flag overexpression plasmid or Vec control were detected using the Western blotting assay. **(E)** Flow cytometry analysis of DDX41-OE and Vec melanoma cells apoptosis induced by 0.5 μg/mL DTIC plus Remodelin. **(F)** Flow cytometry analysis of cell apoptosis of ZNF746-OE and Vec melanoma cells induced by 0.5 μg/mL DTIC plus Remodelin. **(G)** Flow cytometry analysis of NAT10-KD cells overexpressed DDX41 apoptosis induced by 0.5 μg/mL DTIC. **(H)** Flow cytometry analysis of NAT10-KD cells overexpressed ZNF746 apoptosis induced by 0.5 μg/mL DTIC. Two-sided *p*-values were determined by two-way ANOVA test; mean ± SD. Data representative of n = 3 biologically independent experiments unless stated otherwise.

To further clarify whether targeting NAT10 benefits overcoming melanoma chemoresistance, we evaluated a selective inhibitor of NAT10, Remodelin (17), in our previously established DR melanoma cells. We found that their sensitivity to DTIC was meaningfully rescued by Remodelin, as evidenced by the markedly decreased IC_50_ value (**Figure 5A**) and the remarkably augmented apoptotic cells (**Figure 5B**), as well as the accumulated cleavages of PARP as a marker of apoptosis (**Figure 5C**). We further constructed DDX41-overexpressing (DDX41-OE) and ZNF746-overexpressing (ZNF746-OE) melanoma cells to evaluate whether anti-melanoma effects of Remodelin are mediated by DDX41 and ZNF746. Intriguingly, we found that when DDX41 or ZNF746 expression was forcibly expressed (**Figure 5D**), the anti-melanoma effects of Remodelin were largely diminished in DDX41-OE (**Figure 5E**) and ZNF746-OE cells (**Figure 5F**). Meanwhile, the anti-melanoma effects induced by NAT10-knockout were also diminished in overexpressed DDX41 and ZNF746 levels in NAT10-KD cells (**Figures 5G, H**). These results strongly suggest that NAT10 regulates the chemosensitivity of melanoma by modulating the DDX41 and ZNF746 expression.

**Figure 6:**
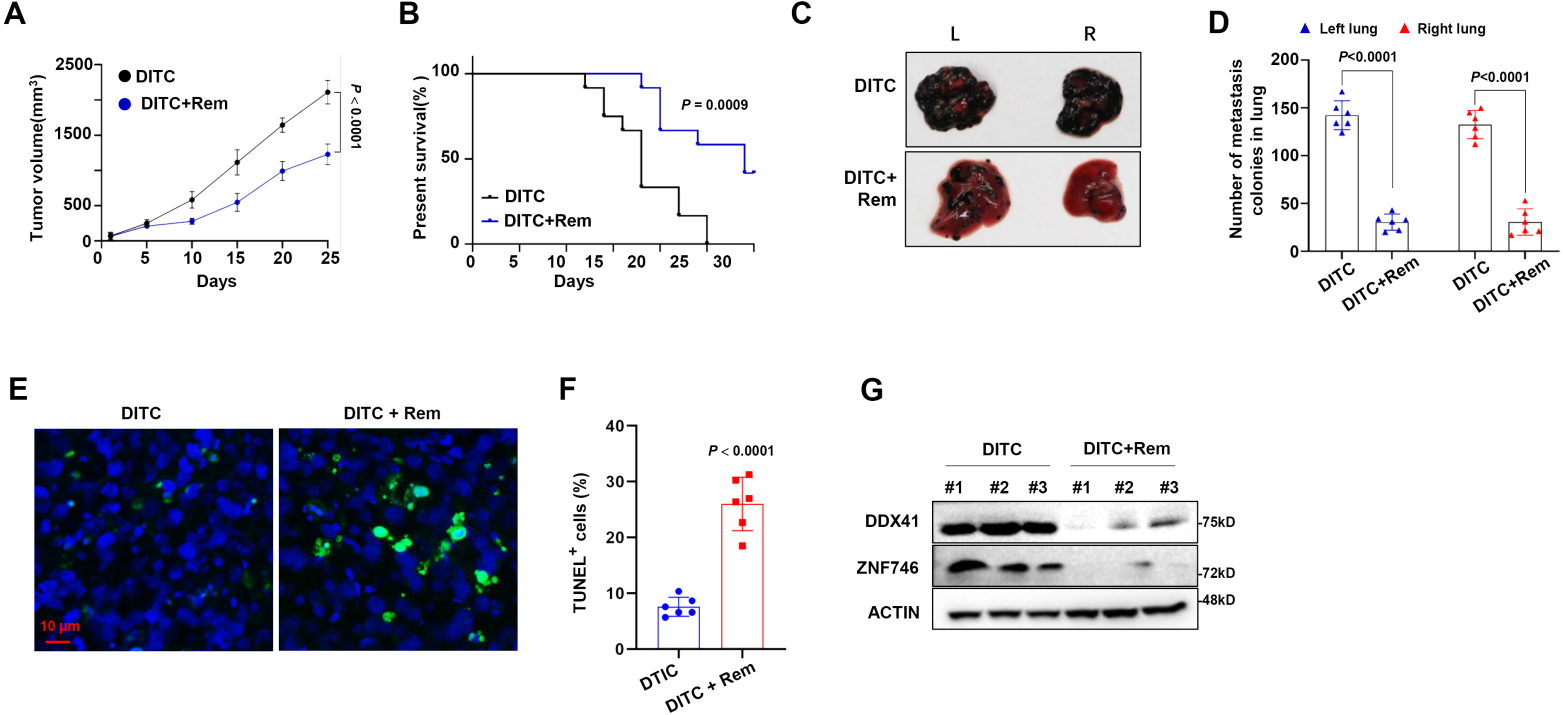
Synergetic effect of dacarbazine (DTIC) and Remodelin in mouse melanoma model. **(A)** A total of 1 × 10^6^ DTIC-resistant (DR) A375 cells were injected into the left flank of mice and treated with 0.5 mg/kg DTIC with or without 0.5 mg/kg Remodelin starting on day 5 twice a week, and tumor volumes were monitored every 3 days (n = 6/group). **(B)** Overall survival (OS) of mice was analyzed using Kaplan–Meier survival analysis. **(C)** Representative photographs of the lungs showing metastases by A375 at 25 days. **(D)** Quantification of total lung mass. **(E)** Confocal image of representative immunofluorescent staining for tumor tissue: TUNEL (Alexa Fluor 488, green) and nuclei (DAPI, blue) at 25 days. **(F)** Quantification of TUNEL-positive cells. **(G)** Western blotting shows the DDX41 and ZNF746 protein levels in DTIC group and DTIC+Rem group. Two-sided *p*-values were determined by two-way ANOVA test; mean ± SD. Data representative of n = 3 biologically independent experiments unless stated otherwise.

Finally, we clarified the significance of targeting NAT10 in overcoming DTIC resistance of melanoma *in vivo*. Notably, NOD/SCID mice bearing DR melanoma cells in a xenograft model showed that when mice were treated with DTIC and Remodelin collaboratively, tumor growth was remarkably suppressed compared with individual administration of DTIC (**Figure 6A**), and the survival rate of mice was significantly prolonged (**Figure 6B**). At the same time, DR melanoma cells were also intravenously injected into the NOD/SCID mice for lung metastases. Mice in the DTIC-treated group still developed pronounced metastatic tumors, but mice in the combination of DTIC and Remodelin-treated group were largely absent from metastatic tumors in the lung (**Figures 6C, D**), clearly indicating that Remodelin significantly decreased lung metastases of DR melanoma cells. Moreover, when tissues of metastatic tumors were stained with TUNEL for apoptosis assay, we also observed that a combination of DTIC and Remodelin induced more noticeable apoptosis than DTIC alone (**Figures 6E, F**). Furthermore, we separated tumor cells from mouse models and found that the DTIC+Rem group had lower protein levels of DDX41 and ZNF746 compared with the DTIC group (**Figure 6G**).

Collectively, these data strongly indicate that targeting NAT10 definitely benefits overcoming the DR of melanoma cells *in vivo*.

## Discussion

Currently, DTIC is the first-line agent approved by the Food and Drug Administration (FDA) for the treatment of melanoma, although the development of chemoresistance remains a hurdle in clinical practice. In this study, we disclosed an important role of NAT10 in DTIC resistance of melanoma, mechanistically through promoting NAT10-mediated ac4C modification of *DDX41* and *ZNF746* mRNAs. Notably, our study clarifies the synergistic anti-melanoma effects of NAT10-specific inhibitor Remodelin with DTIC and sheds light on developing new strategies to improve treatment efficacy for melanoma patients.

The participation of NAT10 in tumorigenesis and tumor progression is well-documented, and its role in drug resistance is increasingly recognized (10). As an acetyltransferase, NAT10 directly acetylates proteins involved in the initiation and progression of cancers (16). For example, NAT10 promotes a mediator for DNA damage checkpoint activation, MORC2 K767Ac, and thus is considered a promising target for sensitizing breast cancer cells to DNA-damaging chemotherapy and radiotherapy (18). Notably, in mammals, NAT10 is the only known RNA acetyltransferase (writer) and is responsible for the ac4C production within a broad range of mRNAs (7). The study also discloses that cisplatin promotes NAT10 accumulation, which stabilizes AHNAK mRNA, and AHNAK-mediated DNA damage repair promotes cisplatin resistance (10). However, the role of NAT10 in melanoma chemoresistance remains to be clarified. We identified increased NAT10 expression in drug-resistant melanoma, which correlates with DTIC sensitivity in melanoma cell lines. In addition, NAT10 expression is associated with melanoma progression patients, and a significant negative correlation of NAT10 with clinical outcomes is also revealed.

Further, we elaborate that the role of NAT10 in promoting melanoma chemoresistance is attributed to its RNA acetyltransferase activity. We detected mRNA acetylation levels in NAT10-OE A375 melanoma cells, and the acRIP-seq delineated the detailed mechanism of how NAT10 promoted melanoma drug resistance. We found that C2H2-ZF family members *DDX41* and *ZNF746* were potential targets of NAT10. C2H2-ZF is the major class of zinc finger protein (ZFP), as well as the largest transcriptional/transcription-regulatory factor family in mammalian cells (19, 20). Increasing lines of evidence have revealed the potential roles of C2H2-ZF in cancer progression, possibly by binding specific DNA sequences to modulate complicated transcriptional networks (21). Amounts of C2H2-ZFs can trigger epithelial–mesenchymal transition (EMT), invasion, and metastasis of cancer (22). However, the underlying mechanisms of C2H2-ZF in cancer progression vary in different cancer types and even in the same cancer type under different types of stress (23). For example, ZNF217 has been found to suppress downstream gene expression by interacting with co-repressors (24). However, some ZNF proteins work as transcriptional activators by interacting with co-activators, including CBP/p300 and C/EBP (25, 26). The post-translational modifications (PTMs) of ZNFs could also affect C2H2-ZF function and gene expression (23). DDX41, a member of the DEAD-box helicase family, is primarily recognized for its role in RNA processing, including splicing, translation initiation, and ribosome assembly (27). Mutations and dysregulation of DDX41 have been reported to be associated with familial myelodysplasia syndrome (MDS)/acute myeloid leukemia (AML) (28). These mutations often lead to impaired DNA damage repair mechanisms and disrupted cell cycle control, contributing to oncogenic transformation and tumor progression (29). ZNF746 is a zinc finger protein that functions as a transcriptional repressor. Recent research has uncovered its role in cancer biology. Dysregulated expression of ZNF746 can lead to altered transcriptional programs that favor tumorigenesis, making it a potential biomarker and therapeutic target in various cancers (30). Intriguingly, ac4C peaks on *DDX41* and *ZNF746* of melanoma cells were abolished upon NAT10 knockdown. The following studies confirmed the correlation between *DDX41* and *ZNF746* expression and melanoma cell drug resistance as well as disease progression in patients. To summarize, our study provides novel mechanistic insights into the mechanism underlying C2H2-ZF family member regulation and also provides new knowledge for understanding how mRNA ac4C modification promotes chemoresistance of melanoma.

Due to the multiplicity of mechanisms, combination therapies have proven effective in optimizing treatment effects. Serious Phase III clinical trials of combination treatment changed the therapeutic landscape in melanoma (31). Based on these findings, vemurafenib and dabrafenib were approved by the FDA for the treatment of unresectable or metastatic melanomas carrying the *BRAF* V600E mutation (32). Thus, an urgent need exists for new therapeutics with a mechanism of action that is fundamentally different from the current therapy. NAT10 inhibition is a mechanistically unique strategy that may have the potential to overcome cancer drug resistance (10). This prompted us to further examine the synergic efficacy of NAT10-specific inhibitor Remodelin and DTIC against melanoma. Indeed, Remodelin could recover the DTIC sensitivity of DR melanoma cells *in vitro* and *in vivo*. Thus, our study clarified the possible combinational application of Remodelin and DTIC in the treatment of melanoma patients. In addition, downstream targets of NAT10 could be used as potential biomarkers for melanoma malignancy before chemoresistance and metastasis, and the combination regimen could be considered for use in patients before disease progression, thus highlighting the translational value of our study.

However, our study has limitations. First, the ac4C “reader” in melanoma cells should be identified in further studies. Second, whether other C2H2-ZF members are involved in NAT10-induced chemoresistance and whether they recruit different interacting proteins, including transcription co-activators/co-repressors, chromatin modifiers, and other transcription factors, also remain to be clarified.

## Conclusion

In summary, our data disclose the role of NAT10 in DTIC resistance of melanoma cells, elucidate the machinery through mediating ac4C modification of *DDX41* and *ZNF746* mRNAs, and shed light on developing new therapeutic strategies using Remodelin and DTIC for melanoma treatment.

## Data Availability

The data presented in the study are deposited in the Gene Expression Omnibus database, accession number GSE269940.
