# Supplementary material for: Acetylcytidine modification of DDX41 and ZNF746 by N-acetyltransferase 10 contributes to chemoresistance of melanoma
Source: Front Oncol. 2024 Aug 23;14:1448890. doi: 10.3389/fonc.2024.1448890 (PMC11377236; doi:10.3389/fonc.2024.1448890)
Supplement: Supplementary file 1 [file DataSheet1.docx]

**Supplementary Method**

**Cell lines**

Human melanoma cell lines MeWo and A375 were purchased from ATCC (American Type Culture Collection, Manassas, VA, USA) and mycoplasma-free substantiated. MTS assay to detect cell viability were performed as previously described[^14^](#_ENREF_14)^,^ [^15^](#_ENREF_15). HEK293T cell was a kind gift from Prof. Yupeng Chen at Department of Biochemistry and Molecular Biology, Tianjin Medical University. Tumor cells were cultured in RPMI-1640 media supplemented with 10% of fetal bovine serum (Gibco, Life Technologies, Carlsbad, CA, USA). The HEK293T cells were cultured in DMEM-high glucose media with 10% fetal bovine serum (Gibco, Life Technologies, Carlsbad, CA, USA). These cells were all cultured at 37°C in a humidified incubator with 5% CO_2_ (Gibco, Life Technologies, Carlsbad, CA, USA). All cells were STR authenticated (Biowing Biotech, Shanghai, China) and mycoplasma-free confirmed with the Universal Mycoplasma Detection Kit (ATCC, Manassas, VA, USA).

**Transfection, virus package, infection and luciferase assay**

HEK-293T cells were transfected using polyethyleneimine (PEI) (Polysciences, Warrington, PA, USA) in the OPTI-MEM medium (Life Technologies, Carlsbad, CA, USA) with a ratio of 1:4 to 1:6 of DNA: PEI. Viral particles were produced by HEK293T cells in a 10 cm dish transfected with 4 μg PMD2G and 6 μg PSPAX2 packaging plasmids, together with 8 μg lentiviral expressing vectors. Supernatant carrying the viral particles was harvested after transfection and concentrated to 1/100 volume by Poly (ethylene glycol) 8,000 (Sigma-Aldrich, St. Louis, MS, USA). For viral infection, melanoma cells were seeded in 6-well-plate after which an increasing dosage of viral concentration and 8 μg/mL polybrene was added, 12 h after infection, the medium was changed and cells were cultured for another 48 h for further experiment.

**Real-time PCR**

Using Trizol (Life Technologies, South San Francisco, CA USA) to isolate total RNA from cells according to the manufacturer's instructions. Total RNA was reverse transcribed using the 5× All-In-One reverse transcription MasterMix (abm, Vancouver, Canada). Quantitative real-time PCR was performed by mixing cDNA, gene-specific primers and EvaGreen 2× qPCR MasterMix (abm, Vancouver, Canada) in the QuantStudio 3 Real-Time PCR System (Applied Biosystems). The primers used in qPCR are listed in **Supplementary resources.**

**Cell viability assays.**

For Cell Counting Kit-8 (CCK-8, APExBIO Technology) assays, cells were seeded at 1 × 10^5^ cells/well in 96-well plates, after which administrated with DTIC, Remodelin or DMSO and incubated at 37 °C in 5 % CO2 for 48 hours. CKK8 reagent was added to each well according to instructions and incubated for 1 hours prior to reading absorbance at 450 nm. percentage = OD value of the treatment group/OD value of the control group × 100.

**Tunnel assay**

Tunnel assay was performed using the DeadEnd™ Fluorometric TUNEL System (Promaga, Tokyo, Japan). For paraffin-embedded sections, wash 3 times by 100% ethanol for 15 minutes at room temperature and then rehydrate samples by sequentially immersing the slides through graded ethanol washes (95%, 85%, 70% and 50%) for 3 minutes each at room temperature. Further wash the slides in PBS 3 times at room temperature and incubate slides with 100μl of the 20μg/ml Proteinase K for 30 minutes at room temperature. Then they were washed and incubated with rTdT incubation buffer at 37°C for 60 minutes in the dark. The samples were washed and stained by DAPI for 5 minutes at room temperature in the dark. Then the samples were washed three times and analyzed by the Olympus FV1000 IX81-SIM Confocal Microscope (Olympus, Tokyo, Japan).

**Western Blotting**

Cells were placed in a culture dish on ice and washed with ice-cold PBS, after which ice-cold RIPA-buffers were added with protease inhibitors (Roche, Indianapolis, IN, USA). Ice cold temperatures were maintained at 4°C for 30 min after which the cells were centrifuged in a microcentrifuge for 20 min at 12,000 rpm at 4°C. A small volume of lysate was removed to perform a protein quantification assay and to adjust the sample concentration. Equal amounts of protein were loaded into the wells of the SDS-PAGE gel, along with the molecular weight marker. the gel ran for 1–2 h at 100 V, transferring the protein from the gel to the nitrocellulose membranes (Pall Corporation, Washington, NY, USA). The membrane was blocked for 1 h at room temperature and then incubated with indicated antibody in blocking buffer at 4°C overnight. Then the membrane was washed in PBST for 3 times and incubated with horseradish peroxidase-conjugated secondary antibodies at room temperature for 1 hour, and finally were visualized using an enhanced chemiluminescence system (Millipore, Los Angeles, CA USA). The representative Western blot images for at least three independent experiments shown in the figures have been cropped and auto contrasted. Quantifications of Western blots were analyzed using Image J Version 1.53c (National Institutes of Health).

**Immunohistochemistry**

3% H_2_O_2_ solution was used to block deparaffinized tissue slides and a 10 melanoma citrate buffer (pH 6.0) was used to retrieve antigen. After successful blocking of the deparaffinized tissue, appropriately diluted primary antibodies were added onto the slides and incubated in a humidified chamber at 4°C overnight, after which diluted biotinylated secondary antibody were incubated for 1 hour at room temperature. DAB substrate solution (Dako, K5361), which was newly made just before use, was utilized to reveal the color of antibody staining. Hematoxylin staining was used to localize Nuclei 1 to 2 min before mounting and capture.

**Flow cytometry analysis** **and cell viability assays.**

Melanoma cells were incubated in 6-well plates and administered with DTIC with or without Remodelin, then cells were collected and follow by washed twice by PBS, apoptosis assay was then carried out using the Annexin V-FITC Apoptosis Detection Kit (Sigma-Aldrich, St. Louis, MO, USA) according to the manufacturer’s instructions. A total of 1×10^6^ cells were stained with 10 μL Annexin V-FITC and 2 μL of PI in the dark to ensure the population abundance. Analysis was performed on CellQuest 3.0 software (BD Biosciences, New Jersey, USA), and interpreted using FlowJo software (Treestar).
